# The impact of *Angiopoietin-2* genetic polymorphisms on susceptibility for malignant breast neoplasms

**DOI:** 10.1038/s41598-022-18712-9

**Published:** 2022-08-25

**Authors:** Gui-Nv Hu, Yan Wang, Chih-Hsin Tang, Lu-Lu Jin, Bi-Fei Huang, Qian Wang, Jun-Kang Shao, Chao-Qun Wang, Chen-Ming Su

**Affiliations:** 1 Department of Surgical Oncology, Affiliated Dongyang Hospital of Wenzhou Medical University, Dongyang, Zhejiang China; 2 Department of Medical Oncology, Affiliated Dongyang Hospital of Wenzhou Medical University, Dongyang, Zhejiang China; 3 Department of Pharmacology, School of Medicine, China Medical University, Taichung, Taiwan; 4 Chinese Medicine Research Center, China Medical University, Taichung, Taiwan; 5 Department of Biotechnology, College of Health Science, Asia University, Taichung, Taiwan; 6 Department of Biomedical Sciences Laboratory, Affiliated Dongyang Hospital of Wenzhou Medical University, Dongyang, Zhejiang China; 7 Department of Pathology, Affiliated Dongyang Hospital of Wenzhou Medical University, Dongyang, Zhejiang 322100 China; 8 Department of Sports Medicine, China Medical University, Taichung, 406040 Taiwan

**Keywords:** Breast cancer, Genetic linkage study, Genotype, Haplotypes

## Abstract

Breast cancer causes morbidity and mortality among women worldwide, despite much research illuminating the genetic basis of this disease. Anti-angiogenesis therapies have been widely studied, although the association between angiopoietin-2 (*ANGPT2*) single nucleotide polymorphisms (SNPs) and breast cancer subtypes remains unclear. This case–control study included 464 patients with malignant breast neoplasms and 539 cancer-free females. We explored the effects of *ANGPT2* SNPs on the susceptibility for a malignant breast neoplasm in a Chinese Han population. Five *ANGPT2* SNPs (rs2442598, rs734701, rs1823375, 11,137,037, and rs12674822) were analyzed using TaqMan SNP genotyping. Carriers of the variant GG allele of rs1823375 were less likely than wild-type carriers to be diagnosed with clinically staged breast cancer, while females with human epidermal growth factor receptor 2 (HER2)-enriched disease carrying the CG or the CG+GG genotype at rs1823375 were significantly less likely than CC genotype carriers to be of lymph node status N1–N3. We also found that the T-T-C-A-T *ANGPT2* haplotype significantly increased the risk for developing a malignant breast neoplasm by 1.385-fold (95% CI: 1.025–1.871; *p* < 0.05). Our study is the first to document a correlation between *ANGPT2* polymorphisms and the development and progression of a malignant breast neoplasm in females of Chinese Han ethnicity.

## Introduction

The latest GLOBOCAN estimates of cancer incidence have revealed that female breast cancer is the most commonly diagnosed cancer worldwide, with around 2.3 million cases in 2020, which equates to 11.7% of all cancer cases^1^. Thus, female breast cancer is a critical component of the global health burden and this disease is particularly problematic for China, because Chinese women have the highest age-standardized rates of cancer incidence worldwide (39.10 per 100,000 population)^1,2^ and breast cancer was the most commonly diagnosed type of tumor in women living in China in the year 2015^2,3^.

Despite recent improvements in 5-year patient survival rates (from 73% between 2003–2005 to 82% in 2012–2015)^4^, increasing numbers of women who are detected early in the disease process, as well as increased access to effective treatments for breast cancer and greater amounts of government funding for cancer control^1,3,4^, breast cancer remains a major health issue among Chinese women^3^. The current situation regarding the high burden of malignant breast neoplasms in China leaves much room for improvement.

The precise etiology of breast cancer remains unclear, although the main risk factors have been identified as female gender, older age and having a family history of breast cancer^5^. Several genome-wide association studies have identified gene-environment interactions and breast cancer susceptibility single nucleotide polymorphisms (SNPs) that are associated with breast cancer risk^6,7^. Thus, exploring risk-associated SNPs may help to estimate individual risks for developing breast cancer and assist with its earlier diagnosis.

Angiopoietin-2 (*ANGPT2*) is a protein coding gene found on chromosome 8 that is capable of serving as a permissive angiogenic signal in the presence of the angiogenic inducer, vascular endothelial growth factor (VEGF), as this combination may facilitate endothelial cell migration and proliferation, whereas in the absence of VEGF, *ANGPT2* induces endothelial cell apoptosis and subsequent vascular regression^8,9^. Interestingly, a case–control study involving Turkish patients with gynecological cancers found no evidence in support of DNA sequence variations in *VEGF* and *ANGPT2* genes and their contribution to ovarian, cervical and endometrial cancers^10^. In contrast, in a study involving 421 critically ill patients with acute kidney injury (AKI) who were of European ancestry, a genetic variant (rs2920656) near the *ANGPT2* gene significantly lowered the risk of developing the AKI sub-phenotype AKI-SP2 and lowered plasma Ang2 levels^11^. In a study that enrolled Chinese Han subjects, those who had the *ANGPT2* SNP rs12674822 were at increased risk for rheumatoid arthritis^12^, while another investigation that also involved Chinese Han subjects reported that two *ANGPT2* SNPs, rs12674822 and rs11137037, were correlated with the development of lung cancer and its progression^13^. These findings suggest that it is worth exploring a possible correlation between *ANGPT2* gene polymorphisms and breast cancer diagnosis.

This case–control study is based on blood samples obtained from 539 healthy, cancer-free women and from 464 women with malignant breast neoplasms. It sought to determine whether Chinese Han women with *ANGPT2* SNPs are susceptible to developing a malignant breast neoplasm and whether these SNPs correlate with clinical disease status.

## Materials and methods

### Study participants

Between 2013 and 2018, we enrolled 464 female patients (cases) diagnosed with a malignant neoplasm of the breast at Dongyang People's Hospital (Dongyang, Zhejiang, China) and 539 healthy, cancer-free females (controls), each of whom provided one 5-mL blood sample. The control group inclusion criteria specified that the enrolled patients underwent physical examination in the same hospital. A healthy status was defined as an absence of cancer history or other chronic diseases. Fully informed written consent was obtained from all study participants before study entry. The trial was performed in accordance with the principles of the Declaration of Helsinki^14^, Good Clinical Practices, and all local laws. The Ethics Committee of Dongyang People's Hospital approved the study protocol (Approval No.2020-YX-117). Clinicopathological data included age, cigarette smoking status, alcohol consumption, clinical stage, pathological grade, estrogen receptor (ER), progesterone receptor (PR), human epidermal growth factor receptor 2 (HER2) and Ki-67 status. Pathohistological diagnosis relied on the World Health Organization breast tumor classification criteria and tumors were graded by the Scarff-Bloom-Richardson method^15,16^. Malignant breast neoplasms were classified into the following 4 subgroups: Luminal A (ER-positive [+] and/or PR^+^, HER2-negative [−], Ki-67 < 14%); Luminal B (ER^+^ and/or PR^+^, HER2^−^, Ki-67 ≥ 14%, ER^+^ and/or PR^+^, HER2^+^); HER2-enriched (ER^−^, PR^−^, HER2^+^), or as triple-negative breast cancer (TNBC; ER^−^, PR^−^, HER2^−^)^17–19^. A standardized questionnaire at study entry collected sociodemographic data and electronic medical records provided clinicopathological information.

### Selection of *ANGPT2* polymorphisms

The *ANGPT2* SNPs selected for this study were identified from multi-allelic copy number variation (CNV) profiles encompassing the q23 region of chromosome 8 containing *ANGPT2* genes. We screened some of these *ANGPT2* SNPs with minor allele frequencies > 5% from the records held on the 1000 Genome Projects website (https://www.internationalgenome.org/), to ensure the accuracy of our genotyping. Finally, nonsynonymous SNPs rs2442598, rs734701, rs1823375, 11,137,037, and rs12674822 were selected and validated by the Single Nucleotide Polymorphism database (dbSNP) (https://www.ncbi.nlm.nih.gov/snp/), as previously described^20^.

### Genomic DNA extraction and genotyping by real-time PCR

Total genomic DNA was extracted from peripheral blood leukocytes using a QIAamp DNA blood mini kit (Qiagen, CA, USA), according to the manufacturer’s instructions^20^. Extracted DNA was stored at − 20 °C and prepared for genotyping by polymerase chain reaction (PCR). DNA was dissolved in TE buffer (10 mM Tris pH 7.8, 1 mM EDTA) and stored at − 20 °C until quantitative PCR analysis. Five *ANGPT2* SNP probes were purchased from Thermo Fisher Scientific Inc. (USA), and allelic discrimination analysis of *ANGPT2* SNPs was assessed using a QuantStudio™ 5 Real-Time PCR system (Applied Biosystems, CA, USA), according to the manufacturer’s instructions. Data were further analyzed with QuantStudio™ Design & Analysis Software (Applied Biosystems)^21–23^. Genotyping PCR was carried out in a total volume of 10 μL containing 20–70 ng genomic DNA, 1 U Taqman Genotyping Master Mix (Applied Biosystems) and 0.25 μL probes. The protocol included an initial denaturation step at 95 °C for 10 min, followed by 40 cycles of 95 °C for 15 s and 60 °C for 1 min.

### Statistical analysis

Testing for Hardy–Weinberg equilibrium was performed to examine the genotypic frequencies of *ANGPT2* rs2442598, rs734701, rs1823375, rs11137037 and rs12674822 in the cancer-free controls (2 degrees of freedom [df2]). Between-group differences were considered significant if *p*-values were less than 0.05. Since the data were independent and normally distributed, chi-square analysis or the Fisher’s exact test was used to compare relationships between *ANGPT2* SNPs and clinicopathological parameters. Odds ratios (ORs) with their 95% confidence intervals (CIs) calculated associations between genotype frequencies and the risk of a malignant breast neoplasm. To further exclude the impact of confounding variables, adjusted odds ratios (AORs) with 95% CIs were estimated by multiple logistic regression models that controlled for age. Analysis of variance (ANOVA) was used to compare the age distribution of the cases and controls, and the Scheffé test was applied for post hoc analysis. The linkage disequilibrium (LD) haplotype analysis was observed using Haploview software (version 4.2) analysis.

## Results

All of the study participants were of Chinese Han ethnicity. Table 1 presents their demographic characteristics and clinical parameters. The mean age was 55.05 ± 11.75 years in the breast cancer cohort and 52.89 ± 11.36 years in the controls (*p* < 0.05). The majority of study participants did not smoke cigarettes or consume alcohol; among those who did, smoking was significantly more common in the control cohort than in the case cohort (2.2% vs 0.2%; *p* < 0.05), while alcohol consumption was more common among cases than controls (7.1% vs 3.9%; *p* > 0.05). Most cases were diagnosed with clinical stage I/II disease (78.7%), but pathological grade II/III (90.3%). Tumors were mostly ER^+^ (71.8%), PR^+^ (59.7%), or HER2^–^ (66.6%) (Table 1).

**Table 1:** Demographic and clinicopathological characteristics of healthy controls and patients with malignant breast neoplasms.

Variable	Controls N = 539 (%)	Patients N = 464 (%)	*p* value
**Age (years)**	**Mean ± S.D**	**Mean ± S.D**	
	40.43 ± 15.62	52.89 ± 11.36	*p* < 0.05
**Cigarette smoking**			
No	527 (97.8)	463 (99.8)	
Yes	12 (2.2)	1 (0.2)	*p* < 0.05
**Alcohol consumption**			
No	518 (96.1)	431 (92.9)	
Yes	21 (3.9)	33 (7.1)	*p* > 0.05
**Clinical stage**			
I + II		365 (78.7)	
III + IV		99 (21.3)	
**Tumor size**			
< T2		215 (46.3)	
≧T2		249 (53.7)	
**Lymph node status**			
N0		255 (55.0)	
N1 + N2 + N3		209 (45.0)	
**Distant metastasis**			
M0		452 (97.4)	
M1		12 (2.6)	
**Pathological grade**			
I		45 (9.7)	
II + III		419 (90.3)	
**ER status**			
Negative		131 (28.2)	
Positive		333 (71.8)	
**PR status**			
Negative		187 (40.3)	
Positive		277 (59.7)	
**HER2 status**			
Negative		309 (66.6)	
Positive		155 (33.4)	
**Ki-67**			
≤ 14%		136 (29.3)	
> 14%		328 (70.7)	

The Mann–Whitney *U* test or Fisher’s exact test was used to compare values between controls and patients with breast cancer. ER, estrogen receptor; PR, progesterone receptor; HER2, human epidermal growth factor receptor 2. Pathological grade: I, well differentiated; II, moderately differentiated; III, poorly differentiated.

*ANGPT2* genotyping results of distribution frequencies for all blood samples are listed in Table 2. The genotypic frequency of the *ANGPT2* genetic variant rs2442598 conformed to the Hardy–Weinberg equilibrium (χ^2^ value, 0.386, *p* = 0.825; df2) in controls. The frequencies of rs734701, rs1823375, rs11137037 and rs12674822 also satisfied the Hardy–Weinberg equilibrium (χ2 value, 4.679, *p* = 0.096; χ2 value, 0.889, *p* = 0.641; χ2 value, 0.494, *p* = 0.781 and χ2 value, 2.473, *p* = 0.290; respectively). In cases and controls, most of those with the rs2442598 SNP and rs11137037 SNP were homozygous for the AA genotype, most of those with the rs734701 SNP were homozygous for the TT genotype, most of those with the variant rs1823375 were homozygous for CC, while most of those with the variant rs12674822 were homozygous for GG (Table 2). Distributions of *ANGPT2* variants rs2442598, rs734701, rs1823375, rs11137037 and rs12674822 did not differ significantly between cases and controls, even after controlling for age (Table 2).

**Table 2:** Distribution frequencies of *ANGPT2* genotypes in controls and patients with malignant breast neoplasms.

Variable	Controls N = 539 (%)	Patients N = 464 (%)	OR (95% CI)	AOR (95% CI)
**rs2442598**				
AA	139 (25.8)	124 (26.7)	1.00 (reference)	1.00 (reference)
AT	287 (53.2)	238 (51.3)	0.930 (0.691–1.251)	0.906 (0.670–1.225)
TT	113 (21.0)	102 (22.0)	1.012 (0.705–1.452)	1.016 (0.704–1.465)
AT+TT	400 (74.2)	340 (73.3)	0.953 (0.719–1.263)	0.935 (0.701–1.246)
**rs734701**				
TT	179 (33.2)	154 (33.2)	1.00 (reference)	1.00 (reference)
TC	247 (45.8)	236 (50.9)	1.111 (0.840–1.469)	1.107 (0.832–1.474)
CC	113 (21.0)	74 (15.9)	0.761 (0.529–1.095)	0.756 (0.523–1.092)
TC+CC	360 (66.8)	310 (66.8)	1.001 (0.769–1.303)	0.996 (0.761–1.302)
**rs1823375**				
CC	260 (48.2)	210 (45.3)	1.00 (reference)	1.00 (reference)
CG	234 (43.4)	213 (45.9)	1.127 (0.869–1.461)	1.143 (0.877–1.489)
GG	45 (8.3)	41 (8.8)	1.128 (0.712–1.788)	1.093 (0.685–1.744)
CG+GG	279 (51.8)	254 (54.7)	1.127 (0.879–1.446)	1.134 (0.880–1.461)
**rs11137037**				
AA	254 (47.1)	211 (45.5)	1.00 (reference)	1.00 (reference)
AC	198 (36.7)	171 (36.9)	1.040 (0.790–1.368)	1.041 (0.786–1.378)
CC	87 (16.1)	82 (17.7)	1.135 (0.797–1.614)	1.173 (0.821–1.675)
AC+CC	285 (52.9)	253 (54.5)	1.069 (0.833–1.371)	1.086 (0.842–1.399)
**rs12674822**				
GG	166 (30.8)	122 (26.3)	1.00 (reference)	1.00 (reference)
GT	248 (46.0)	227 (48.9)	1.245 (0.927–1.673)	1.191 (0.880–1.610)
TT	125 (23.2)	115 (24.8)	1.252 (0.887–1.767)	1.208 (0.853–1.710)
GT+TT	373 (69.2)	342 (73.7)	1.248 (0.947–1.644)	1.192 (0.900–1.578)

The odds ratios (ORs) and their associated 95% confidence intervals (CIs) were estimated by logistic regression analysis. The adjusted odds ratios (AORs) with their associated 95% CIs were estimated by multiple logistic regression analysis that controlled for age. *p < 0.05 was considered to be statistically significant.

Next, *ANGPT2* alleles in patients with malignant breast neoplasms were examined to clarify the role of *ANGPT2* polymorphisms in clinical stage, primary tumor size, lymph node metastasis, distant metastasis, and pathological grade. For the 464 patients with malignant breast neoplasms, a significant correlation between rs1823375 homozygous variants (CC vs GG) and clinical stage (OR: 0.274, 95% CI: 0.081–0.927, *p* < 0.05) was observed, which persisted when estimated by multiple logistic regression models (AOR: 0.255, 95% CI: 0.074–0.876, *p* < 0.05) (Table 3).

**Table 3:** Odds ratios (ORs) and 95% confidence intervals (CIs) of clinical status and *ANGPT2* rs1823375 genotypic frequencies in 464 patients with malignant breast neoplasms.

Gene Genotypes	Patients		OR (95% CI)	AOR (95% CI)
	Clinical stage			
	Stage I/II	Stage III/IV		
rs1823375	N = 365 (%)	N = 99 (%)		
CC	163 (44.7)	47 (47.5)	1.00 (reference)	1.00 (reference)
CG	164 (44.9)	49 (49.5)	1.036 (0.657–1.633)	1.104 (0.751–1.622)
GG	38 (10.4)	3 (3.0)	**0.274 (0.081**–0.927)*	**0.255 (0.074**–0.876)*
CG+GG	202 (55.3)	52 (52.5)	0.893 (0.572–1.393)	1.077 (0.746–1.557)

Gene Genotypes	Patients		OR (95% CI)	AOR (95% CI)
	Tumor size			
	< T2	≧T2		
rs1823375	N = 215 (%)	N = 249 (%)		
CC	100 (46.5)	110 (44.2)	1.00 (reference)	1.00 (reference)
CG	95 (44.2)	118 (47.4)	1.129 (0.770–1.655)	1.104 (0.751–1.622)
GG	20 (9.3)	21 (8.4)	0.955 (0.489–1.865)	0.928 (0.473–1.824)
CG+GG	115 (53.5)	139 (55.8)	1.099 (0.762–1.585)	1.077 (0.746–1.557)

Gene Genotypes	Patients		OR (95% CI)	AOR (95% CI)
	Lymph node metastasis			
	N0	N1 + N2 + N3		
rs1823375	N = 255 (%)	N = 209 (%)		
CC	112 (43.9)	98 (46.9)	1.00 (reference)	1.00 (reference)
CG	118 (46.3)	95 (45.5)	0.920 (0.627–1.349)	0.908 (0.618–1.334)
GG	25 (9.8)	16 (7.7)	0.731 (0.369–1.449)	0.707 (0.355–1.407)
CG+GG	143 (56.1)	111 (53.1)	0.887 (0.614–1.281)	0.870 (0.601–1.259)

Gene Genotypes	Patients		OR (95% CI)	AOR (95% CI)
	Distant metastasis			
	M0	M1		
rs1823375	N = 452 (%)	N = 12 (%)		
CC	204 (45.1)	6 (50.0)	1.00 (reference)	1.00 (reference)
CG	207 (45.8)	6 (50.0)	0.986 (0.313–3.106)	0.983 (0.308–3.142)
GG	41 (9.1)	0 (0)	–	–
CG+GG	248 (54.0)	6 (50.0)	0.823 (0.261–2.589)	0.804 (0.252–2.563)

Gene Genotypes	Patients		OR (95% CI)	AOR (95% CI)
	Pathological grade			
	I	II + III		
rs1823375	N = 320 (%)	N = 144 (%)		
CC	150 (46.9)	60 (41.7)	1.00 (reference)	1.00 (reference)
CG	142 (44.4)	71 (49.3)	1.195 (0.626–2.279)	1.248 (0.650–2.399)
GG	28 (8.8)	13 (9.0)	1.082 (0.352–3.325)	1.065 (0.345–3.290)
CG+GG	170 (53.1)	84 (58.3)	1.175 (0.635–2.175)	1.210 (0.651–2.252)

The ORs with their 95% CIs were estimated by logistic regression models. The adjusted odds ratios (AORs) with their 95% CIs were estimated by multiple logistic regression models that controlled for alcohol consumption and age. *p < 0.05 was considered to be statistically significant.

Significant values are in bold.

We further analyzed the correlation between clinical parameters and rs2442598, rs734701, rs1823375, rs11137037 and rs12674822 genotyping frequencies for the different breast cancer subtypes (Luminal A, Luminal B, HER2-enriched and TNBC) (Table 4). Among patients with the HER2-enriched subtype, those carrying the CG or the CG+GG allele at rs1823375 were significantly less likely than CC allele carriers to be of lymph node status N1–N3 (OR: 0.325; 95% CI: 0.112–0.940 and 0.313; 0.114–0.862, respectively) (Table 4).

**Table 4:** Odds ratios (ORs) and 95% confidence interval (CIs) of *Ang2* genotypic frequencies and clinical subtypes in patients with malignant breast neoplasms.

Variable	Luminal A (n = 128)			Luminal B (n = 206)			HER2 overexpression (n = 68)			TNBC (n = 62)		
	Clinical stage		OR (95% CI)	Clinical stage		OR (95% CI)	Clinical stage		OR (95% CI)	Clinical stage		OR (95% CI)
	Stage I/II	Stage III/IV		Stage I/II	Stage III/IV		Stage I/II	Stage III/IV		Stage I/II	Stage III/IV	
rs1823375	N = 109 (%)	N = 19 (%)		N = 155(%)	N = 51(%)		N = 49 (%)	N = 19 (%)		N = 52 (%)	N = 10 (%)	
CC	51 (46.8)	8 (42.1)	1.00 (reference)	70 (45.2)	24 (47.1)	1.00 (reference)	18 (36.7)	11 (57.9)	1.00 (reference)	24 (46.2)	4 (40.0)	1.00 (reference)
CG	48 (44.0)	10 (52.6)	1.328 (0.484**–**3.646)	69 (44.5)	25 (49.0)	1.057 (0.551–2.027)	23 (46.9)	8 (42.1)	0.569 (0.189–1.710)	24 (46.2)	6 (60.0)	1.500 (0.375–5.998)
GG	10 (9.2)	1 (5.3)	0.638 (0.072**–**5.677)	16 (10.3)	2 (3.9)	0.365 (0.078**–1**.703)	8 (16.3)	0 (0)	–	4 (7.7)	0 (0)	–
CG+GG	58 (53.2)	11 (57.9)	1.209 (0.451**–**3.239)	85 (54.8)	27 (52.9)	0.926 (0.491**–1**.747)	31 (63.3)	8 (42.1)	0.422 (0.143–1.244)	28 (53.8)	6 (60.0)	1.286 (0.324**–5**.098)

Variable	Luminal A (n = 128)			Luminal B (n = 206)			HER2 overexpression (n = 68)			TNBC (n = 62)		
	Tumor size		OR (95% CI)	Tumor size		OR (95% CI)	Tumor size		OR (95% CI)	Tumor size		OR (95% CI)
	< T2	≧T2		< T2	≧T2		< T2	≧T2		< T2	≧T2	
rs1823375	N = 85(%)	N = 43(%)		N = 83(%)	N = 123(%)		N = 22(%)	N = 46(%)		N = 25(%)	N = 37(%)	
CC	43 (50.6)	16 (37.2)	1.00 (reference)	37 (44.6)	57 (46.3)	1.00 (reference)	8 (36.4)	21 (45.7)	1.00 (reference)	12 (48.0)	16 (43.2)	1.00 (reference)
CG	35 (41.2)	23 (53.5)	1.766 (0.811**–**3.847)	37 (44.6)	57 (46.3)	1.000 (0.557–1.795)	12 (54.5)	19 (41.3)	1.603 (0.203–1.792)	11 (44.0)	19 (51.4)	1.295 (0.451–3.718)
GG	7 (8.2)	4 (9.3)	1.536 (0.396**–**5.959)	9 (10.8)	9 (7.3)	0.649 (0.236**–1**.786)	2 (9.1)	6 (13.0)	1.143 (0.190–6.883)	2 (8.0)	2 (5.4)	0.750 (0.092–6.112)
CG+GG	42 (49.4)	27 (62.8)	1.728 (0.816**–**3.659)	46 (55.4)	66 (53.7)	0.931 (0.532**–1**.630)	14 (63.6)	25 (54.3)	0.680 (0.239–1.933)	13 (52.0)	21 (56.8)	1.212 (0.437–3.357)

Variable	Luminal A (n = 128)			Luminal B (n = 206)			HER2 overexpression (n = 68)			TNBC (n = 62)		
	Lymph node status		OR (95% CI)	Lymph node status		OR (95% CI)	Lymph node status		OR (95% CI)	Lymph node status		OR (95% CI)
	N0	N1–N3		N0	N1–N3		N0	N1–N3		N0	N1–N3	
rs1823375	N = 85(%)	N = 43(%)		N = 103(%)	N = 103(%)		N = 32(%)	N = 36(%)		N = 35(%)	N = 27(%)	
CC	39 (45.9)	20 (46.5)	1.00 (reference)	48 (46.6)	46 (44.7)	1.00 (reference)	9 (28.1)	20 (55.6)	1.00 (reference)	16 (45.7)	12 (44.4)	1.00 (reference)
CG	39 (45.9)	19 (44.2)	0.950 (0.440–2.050)	44 (42.7)	50 (48.5)	1.186 (0.669–2.102)	18 (56.3)	13 (36.1)	0.325 (0.112–0.940)*	17 (48.6)	13 (48.1)	1.020 (0.360–2.885)
GG	3 (8.2)	4 (9.3)	1.114 (0.291**–**4.262)	11 (10.7)	7 (6.8)	0.664 (0.237**–1**.861)	5 (15.6)	3 (8.3)	0.270 (0.053**–**1.383)	2 (5.7)	2 (7.4)	1.333 (0.164–10.867)
CG+GG	46 (54.1)	23 (53.5)	0.975 (0.467–2.035)	55 (53.4)	57 (55.3)	1.081 (0.625**–1**.871)	23 (71.9)	16 (44.4)	0.313 (0.114–0.862)*	19 (54.3)	15 (55.6)	1.053 (0.384–2.888)

Variable	Luminal A (n = 128)			Luminal B (n = 206)			HER2 overexpression (n = 68)			TNBC (n = 62)		
	Distant metastasis		OR (95% CI)	Distant metastasis		OR (95% CI)	Distant metastasis		OR (95% CI)	Distant metastasis		OR (95% CI)
	M0	M1		M0	M1		M0	M1		M0	M1	
rs1823375	N = 127(%)	N = 1(%)		N = 201(%)	N = 5(%)		N = 65(%)	N = 3(%)		N = 59(%)	N = 3(%)	
CC	59 (46.5)	0 (0)	1.00 (reference)	90 (44.8)	4 (80.0)	1.00 (reference)	28 (43.1)	1 (33.3)	1.00 (reference)	27 (45.8)	1 (33.3)	1.00 (reference)
CG	57 (44.9)	1 (100.0)	–	93 (46.3)	1 (20.0)	0.242 (0.027–2.206)	29 (44.6)	2 (66.7)	1.931 (0.166**–**22.512)	28 (47.5)	2 (66.7)	1.929 (0.165–22.528)
GG	11 (8.7)	0 (0)	–	18 (9.0)	0 (0)	–	8 (12.3)	0 (0)	**–**	4 (6.8)	0 (0)	–
CG+GG	68 (53.5)	1 (100.0)	–	111 (55.2)	1 (20.0)	0.203 (0.022**–1**.846)	37 (56.9)	2 (66.7)	1.514 (0.131**–**17.542)	32 (54.2)	2 (66.7)	1.688 (0.145–19.643)

Variable	Luminal A (n = 128)			Luminal B (n = 206)			HER2 overexpression (n = 68)			TNBC (n = 62)		
	Pathological grade		OR (95% CI)	Pathological grade		OR (95% CI)	Pathological grade		OR (95% CI)	Pathological grade		OR (95% CI)
	I	II + III		I	II + III		I	II + III		I	II + III	
rs1823375	N = 35(%)	N = 93(%)		N = 7(%)	N = 199(%)		N = 2(%)	N = 66(%)		N = 1(%)	N = 61(%)	
CC	18 (51.4)	41 (44.1)	1.00 (reference)	3 (42.9)	91 (45.7)	1.00 (reference)	1 (50.0)	28 (42.4)	1.00 (reference)	0 (0)	28 (45.9)	1.00 (reference)
CG	15 (42.9)	43 (46.2)	1.259 (0.561–2.823)	3 (42.9)	91 (45.7)	1.000 (0.197–5.086)	1 (50.0)	30 (45.5)	1.071 (0.064**–**17.962)	0 (0)	30 (49.2)	–
GG	2 (5.7)	9 (9.7)	1.976 (0.387–10.076)	1 (14.3)	17 (8.5)	0.560 (0.055–5.712)	0 (0)	8 (12.1)	–	1 (100.0)	3 (4.9)	–
CG+GG	17 (48.6)	52 (55.9)	1.343 (0.616**–**2.927)	4 (57.1)	108 (54.3)	0.890 (0.194**–**4.081)	1 (50.0)	38 (57.6)	1.357 (0.081**–**22.643)	1 (100.0)	33 (54.1)	–

The ORs and their associated 95% CIs were estimated by logistic regression models. *p < 0.05 was considered to be statistically significant. HER2, human epidermal growth factor receptor 2; TNBC, triple-negative breast cancer. Pathological grade: I, well differentiated; II, moderately differentiated; III, poorly differentiated.

Significant values are in bold.

**Figure 1:**
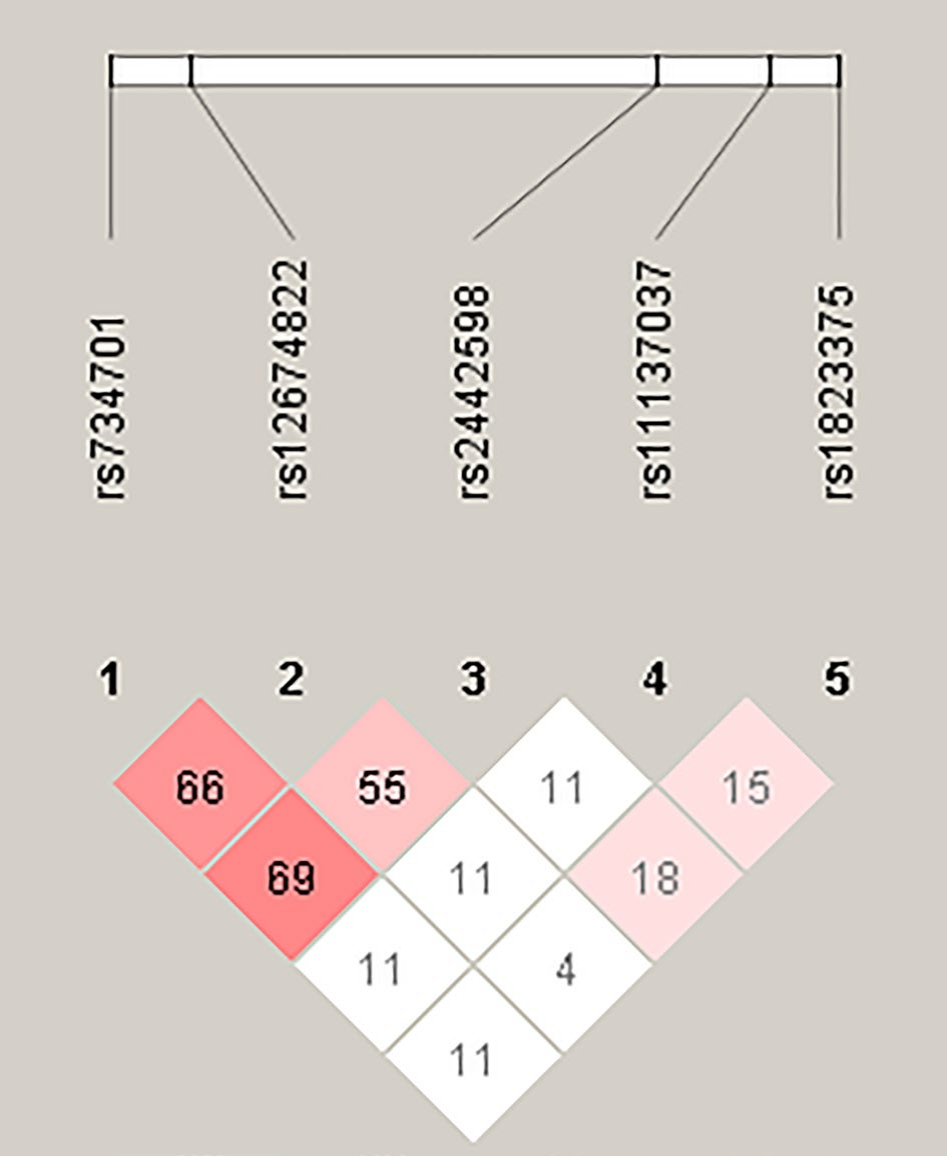
Linkage disequilibrium patterns of five single nucleotide polymorphisms in the *ANGPT2* gene.

We further explored the haplotypes to evaluate the combined effects of the *ANGPT2* polymorphisms on malignant breast neoplasm susceptibility. The most common haplotype in the control cohort was A-C-C-A-G (21.4%), which was therefore chosen as the reference. Compared with the reference, the T-T-C-A-T *ANGPT2* haplotype significantly increased the risk for developing a malignant breast neoplasm by 1.385-fold (95% CI: 1.025–1.871; *p* < 0.05) (Table 5). A reconstructed linkage disequilibrium (LD) plot of the five *ANGPT2* SNPs is shown in Fig. 1. Our reconstructed results illustrate one haploblock in which rs734701 and rs2442598 showed 69% LD (Fig. 1).

**Table 5:** Distribution frequencies of *ANGPT2* haplotypes in healthy controls and patients with malignant breast neoplasms.

Haplotype block					Controls N = 1078 (%)	Patients N = 928 (%)	OR (95% CI)
rs2442598 A/T	rs734701 T/C	rs1823375 C/G	rs11137037 A/C	rs12674822 G/T			
A	C	C	A	G	231 (21.4)	189 (20.4)	Reference
T	T	C	A	T	135 (12.5)	153 (16.5)	1.385 (1.025─1.871)*
T	T	G	A	T	105 (9.7)	108 (11.6)	1.257 (0.904─1.749)
A	C	G	C	G	80 (7.4)	93 (10.0)	1.421 (0.996─2.027)
T	T	C	C	T	82 (7.6)	83 (8.9)	1.237 (0.863─1.774)
A	T	C	C	G	52 (4.8)	56 (6.0)	1.316 (0.862─2.010)
Others^#^					393 (36.6)	246 (26.6)	–

The odds ratios (ORs) and their associated 95% confidence intervals (CIs) were estimated by logistic regression models. *p < 0.05 was considered to be statistically significant. # denotes all other 25 haplotypes without significance.

Significant values are in bold.

## Discussion

Several genome-wide association studies have used genetic SNPs as potential markers for early diagnosis and targeted therapy in breast cancer^24–26^. A recent investigation attributed 18% of the familial risk of breast cancer risk to SNPs^6^, although any potential correlation between *ANGPT2* gene polymorphisms and malignant breast neoplasms has not been explored so far. Previous studies have reported finding similar genetic characteristics in breast tumors for Chinese Han women and women of European ancestry^27,28^. *ANGPT1, ANGPT2* and *FGF2* have been linked in a US study to pathological complete responses to bevacizumab, the first approved recombinant humanized monoclonal antibody for breast cancer^29^, although most investigations into breast cancer genetic polymorphisms have focused on *BRCA1/2*^30,31^. Our previous work found nonsynonymous *ANGPT2* gene polymorphisms in lung and colorectal cancers^13,20^, which suggests that *ANGPT2* SNPs might also be associated with a risk for breast cancer. It is therefore important and appropriate to investigate the impacts of *ANGPT2* genetic variants upon the risk of malignant breast neoplasms among women of Chinese Han ethnicity.

Based on blood samples from 539 healthy, cancer-free women and 464 women with malignant breast neoplasms, we found no significant between-group distribution frequencies for any of the five *ANGPT2* SNPs. We therefore sought to determine whether any of the *ANGPT2* SNPs correlated with clinical status or the pathological grade of the tumors. In analyses adjusted for confounding factors, patients carrying the GG allele at rs1823375 were at lower risk for developing clinically staged breast cancer. Interestingly, our previous research revealed that this variant rs1823375 increased the risk of colorectal cancer in a Chinese Han cohort^20^. A possible explanation for this difference in risk between different cancer types is that gender has disparate effects, but this requires further research.

We also found that the variant rs1823375 lowered the risk of developing lymph node metastasis in patients with HER2^+^ disease. A recent study reported that stromal overexpression of the SNAIL transcription factor SNAI2 is linked to poor prognosis in patients with Luminal B HER2^+^ breast cancer, while the absence of SNAI2 in the stroma of Luminal B HER2^+^ breast tumor cells is associated with lower levels of plasma Ang2^32^. Another study has indicated that *ANGPT2* variants rs2515409 and rs13269021 are associated with pathological complete responses to bevacizumab treatment in breast cancer^29^. Accordingly, *ANGPT2* mRNA levels and survival rates in women with breast cancer deserve more investigation in future clinical trials.

LD in the human genome is critical for genetic variation and features in the detection and treatment of disease^33^. Haplotype analyses can provide significant statistical power for clarifying the contribution of genes to disease susceptibility^34^. To analyze the common haplotypes, we used Haploview software and the PHASE program. As shown in Fig. 1, we identified three LD haploblocks (rs2442598, rs734701 and rs12674822) that were minimally associated with the risk of malignant breast neoplasms; other SNPs were outside the haploblock. Details of the underlying mechanisms require further investigations. When we analyzed the contributions of different haplotype combinations of the five *ANGPT2* variants investigated in this study upon the risk of a malignant breast neoplasm, we found that the TTCAT haplotype increased the risk. It is possible that the *ANGPT2* TTCAT haplotype is in LD with other functional polymorphisms that increase the susceptibility for a malignant breast neoplasm.

The main strength of our study is its systematic analysis of *ANGPT2* SNPs and susceptibility to malignant breast neoplasms in our study population. Potential limitations include the possibility that the study observations merely reflect a cross-sectional relationship instead of actual causality. Moreover, the study participants were all enrolled from only one hospital, which gives rise to possible selection bias. Lastly, our modest sample size requires a larger, independent cohort study to confirm our findings.

## Conclusion

In conclusion, our systematic genotyping results demonstrate an association between *ANGPT2* gene variants and susceptibility for a malignant breast neoplasm and its progression among Chinese Han women. In this study, the *ANGPT2* rs1823375 polymorphism appeared to be protective against clinically staged breast cancer and also lymph node disease, while the T-T-C-A-T ANGPT2 haplotype appeared to significantly increase the risk for developing a malignant breast neoplasm. These findings suggest that the rs1823375 polymorphism can serve as a diagnostic marker for malignant breast neoplasms.

## Data Availability

The data are not publicly available due to the records containing private information of the study participants. However, data are available from the authors upon reasonable request and with permission of Chao-Qun Wang and Chen-Ming Su. The datasets generated and/or analyzed during the current study are available in the 1000 Genome Projects repository (https://www.internationalgenome.org/) and the dbSNP repository (https://www.ncbi.nlm.nih.gov/snp/).
